# The influence of unhealthy lifestyle on semen quality: results from a cross-sectional study and Mendelian randomization analysis

**DOI:** 10.1097/MS9.0000000000004982

**Published:** 2026-04-29

**Authors:** Mengyuan Lin, Yangkun Feng, Yun Zhang, Qingwen He, Ninghan Feng

**Affiliations:** a Center of Reproductive Medicine, Affiliated Women’s Hospital of Jiangnan University, Wuxi, Jiangsu, China; b Wuxi School of Medicine, Jiangnan University, Wuxi, Jiangsu, China; c Department of Public Health, Affiliated Women’s Hospital of Jiangnan University, Wuxi, Jiangsu, China; d Department of Urology, Jiangnan University Medical Center, Wuxi, Jiangsu, China

**Keywords:** cross-sectional study, Mendelian randomization, semen quality, unhealthy lifestyle

## Abstract

**Background and aims:**

Unhealthy lifestyle may influence semen quality. This research aimed to investigate the association of unhealthy lifestyle with the risk of low semen quality and further to examine the causal relationship by Mendelian randomization (MR) method.

**Methods:**

The association of six unhealthy lifestyle habits (smoking, alcohol consumption, coffee consumption, sedentary behavior, obesity, and sleep disturbance) with semen quality was assessed using binary logistic regression analysis including 508 participants. A two-sample MR analysis for seven unhealthy lifestyle habits (smoking, alcohol consumption, caffeine consumption, cannabis consumption, sedentary behavior, obesity, and sleep disturbance) and semen quality was conducted. The principal analysis employed the inverse-variance-weighted (IVW) approach. The MR-pleiotropy residual sum and outlier (MR-PRESSO) tests and MR-Egger regression were implemented to evaluate horizontal pleiotropy. Sensitivity analyses were performed with Cochran’s *Q* test, leave-one-out analysis, and the funnel plot.

**Results:**

During the cross-sectional study, smoking, heavy alcohol and coffee consumption, and sedentary behavior were found to be significantly correlated with an elevated prevalence of low semen quality in the two models (*P* < 0.05). In the IVW of MR analyses, a causal relationship between smoking/alcohol consumption/caffeine consumption/cannabis use/sedentary behavior and the semen quality–related genetic aspects (WFDC3, PATE1, CFAP45, SPEF1, CREM, SPATA20, SPATA9, SPATA46, CCDC103, CRISP2, EQTN, and SPAG11A) was observed (*P* < 0.05). The robustness of the above results was found to be reliable with no pleiotropy. MR sensitivity analyses yielded consistent results.

**Conclusion:**

Our cross-sectional study indicated that smoking, heavy alcohol and coffee consumption, and sedentary behavior were significantly correlated with low semen quality. The MR study supported a causal association between smoking/alcohol consumption/caffeine consumption/cannabis use/sedentary behavior and low semen quality.

## Introduction

Approximately 60–70 million couples, or 15% of the world’s population, are affected by infertility and 40–50% of these cases are related to male factors^[^1,2^]^. A recent meta-analysis including 185 studies and more than 42 000 men without known fertility problems found that human semen quality declined by approximately 50–60% worldwide in the last decade, causing a serious concern on human reproductive health^[^3^]^. The trend seems to be more obvious in particular industrialized and developed countries and might be related to unhealthy lifestyle factors. Numerous studies support the view that preventable unhealthy lifestyle choices, such as smoking^[^4–6^]^, alcohol^[^7,8^]^, caffeine^[^9,10^]^ and cannabis^[^11^]^ consumption, sedentary behavior^[^12,13^]^, obesity^[^14,15^]^, and sleep disturbance^[^16,17^]^, are closely associated with semen quality. A study including 1222 participants with a median age of 23 years found that heavy smoking was associated with the decrease of total sperm count, sperm concentration, and motility, as well as the increase of teratozoospermia^[^5^]^. However, a meta-analysis covering 10 823 infertile male participants (5257 smokers and 5566 non-smokers) showed that sperm motility might not be related to smoking^[^18^]^.

HIGHLIGHTS

- In cross-sectional study, smoking, heavy alcohol and caffeine consumption, and sedentary behavior were significantly correlated with low semen quality.
- In Mendelian randomization analyses, a causal relationship was found between smoking, alcohol, caffeine and cannabis consumption, and sedentary behavior and the semen quality genetic aspects.
- The research method is also suitable to explore the causal relationships between unhealthy lifestyle and other diseases.

Thus, the causal link between unhealthy lifestyle and semen quality has not been fully established by observational studies due to the interference from confounding variables and observation bias. On the other hand, utilizing the single-nucleotide polymorphisms (SNPs) as genetic markers makes it possible to more accurately assess the relationship between exposure and outcome in a defined background. Mendelian randomization (MR) approach provides such an instrument. MR analysis allows us to effectively eliminate the influence of genetic factors and establish a genuine association between the unhealthy lifestyle and semen quality.

This study aimed to investigate the correlation of six unhealthy lifestyle factors such as smoking, alcohol consumption, coffee consumption, sedentary behavior, obesity, and sleep disturbance with semen quality based on observational data. Moreover, using the MR analysis we assessed the associations between unhealthy lifestyle and the semen quality-related genetic aspects. During the MR analysis, seven unhealthy lifestyle habits (smoking, alcohol consumption, caffeine consumption, cannabis consumption, sedentary behavior, obesity, and sleep disturbance) were selected as our exposure variables, and genetic aspects of oligozoospermia, asthenozoospermia, teratozoospermia, and abnormal acrosome were selected as outcomes.

## Methods

### Overall study design and study population

Two stages of analyses were conducted in this study. In stage 1, the clinical data were collected for a cross-sectional study. We performed binary regression analysis to determine the association of smoking, alcohol consumption, coffee consumption, sedentary behavior, obesity, and sleep disturbance with semen quality. In stage 2, we assessed the causal effect of genetically determined levels of seven unhealthy lifestyle habits (smoking, alcohol consumption, caffeine consumption, cannabis consumption, sedentary behavior, obesity, and sleep disturbance) on genetic aspects of semen quality by two-sample MR analysis of summary statistics data from the genome-wide association study (GWAS).

### Cross-sectional study

#### Study subjects

Patients attending the Andrology Clinic and meeting the criteria from January 2024 to December 2024 were included in the study. The inclusion criteria were: (1) the age <50 years and (2) having a need for fertility examination. Exclusion criteria were: (1) family history of hereditary diseases, (2) diagnosis of malignancies in the reproductive system, (3) abnormalities of the testis/epididymis/vas deferens, and (4) infection of the reproductive tract. Each patient included in the study had a physical evaluation and examination. After application of criteria, 508 cases were included for the study. The study was performed according to the guidelines by the Strengthening The Reporting of Cohort Studies in Surgery (STROCSS)^[^19^]^.

#### Semen quality analyses

After abstinence for 2–7 days, semen samples were collected by masturbation. After liquefaction at 37 °C, semen quality analyses were conducted following the criteria of “WHO laboratory manual for the examination and processing of human semen (5th)” as described previously^[^20^]^.

#### Participants’ basic characteristics and lifestyle

The basic characteristics and lifestyle of participants were obtained through questionnaire. Following the criteria, the smoking situation conditions were categorized into non-smoking, former smoking, and current smoking groups^[^21^]^. The body mass index (BMI) situation was divided into obesity, overweight, and normal weight groups^[^22^]^. The alcohol consumption was divided into non-alcohol consumption, former alcohol consumption, mild alcohol consumption, and heavy alcohol consumption groups^[^23^]^. The coffee consumption was divided into non-coffee consumption, mild coffee consumption, and heavy coffee consumption groups^[^24^]^. The sedentary behavior was divided into sedentary time <8 hours/day and sedentary time ≥8 hours/day groups^[^25^]^. The education level was divided into less than or equal to high school, college degree, and higher than or equal to master degree groups. The sleep disturbance was divided into sleep time <6 hours/day, sleep time from 6 to 9 hours/day and sleep time >9 hours/day groups^[^26^]^.

#### Statistical analysis

The R statistical software version 4.4.0 was used to statistically analyze the clinical data. The quantitative data conforming to normal distribution were expressed as mean ± standard deviation (SD). Categorical data were expressed as frequency or percentage. The chi-square tests and unpaired Student’s *t*-tests were applied to categorical data and continuous data for comparison. To evaluate the association between unhealthy lifestyle and semen quality, a binary logistic regression analysis was performed. The odds ratios (OR) and corresponding 95% confidence intervals (CIs) were computed. Age and education level were adjusted in Model 2, and no variables were adjusted in Model 1 in the multivariable analysis. In binary logistic regression analysis, variables with differences were included in the two logistic models. Trend analysis was performed for continuous variable. The diagnostic accuracy of models in detecting low semen quality was assessed by the receiver operating characteristic (ROC) curves as well as the area under the ROC curve (AUC) in different groups. In all analyses, statistical significance was established when the double tailed *P-*value was < 0.05.

### Mendelian randomization

#### Data sources

A comprehensive search in the large-scale GWAS catalog was conducted to find published GWAS data related to genetic aspects of oligozoospermia, asthenozoospermia, teratozoospermia, and abnormal acrosome. Outcome data were mainly derived from two cohorts, with one of the cohorts performing genome-wide testing of 10.6 million imputed autosomal variants against levels of 2994 plasma proteins in 3301 individuals of European descents. Another cohort was obtained from 466 individuals. Smoking and alcohol consumption were performed by the Sequencing Consortium of Alcohol and Nicotine Use, involving 2 669 029 individuals of European ancestry. The data for caffeine consumption were obtained from 263 464 European ancestry individuals, while the data for sedentary behavior came from 408 815 European ancestry individuals (122 996 cases and 289 117 controls). Cannabis consumption and obesity data were available in the IEU Open GWAS Project database. Additionally, GWAS summary statistics for sleep disturbance was extracted from the FinnGen consortium R10 release. Supplemental Digital Content Table S1, available at: http://links.lww.com/MS9/B140 provides details of the GWAS summary level data of exposure and outcome analyzed in this MR study.

#### Selection of genetic instrumental variants

Genetic instruments were extracted from relevant GWAS databases. We included 1752, 501, 19, 13, 188, 35, and 16 independent genetic variants in MR analysis for smoking, alcohol consumption, caffeine consumption, cannabis consumption, sedentary behavior, obesity, and sleep disturbance, respectively, as detailed in Supplemental Digital Content Table S2, available at: http://links.lww.com/MS9/B140. SNPs for cannabis consumption and obesity were identified through GWAS at a significance threshold of *P* < 5 × 10^–6^. SNPs for other exposures were identified using a genome-wide significance threshold of *P* < 5 × 10^–8^. By calculating the *F*-statistics the weak instruments were excluded. The strength of each instrumental variable (IV) was determined, with included IV having an *F*-statistic value >10 in the MR analysis. Including a window size of 10 000 k band linkage disequilibrium (LD) clumping with *r*^2^ < 0.001, the strict criteria were applied. To ensure the specificity of SNPs, the PhenoScanner tool (http://www.phenoscanner.medschl.cam.ac.uk/)^[^27^]^ was employed to validate the exclusion of pleiotropic effects on other phenotypes at the genome-wide significance level, which might have introduced confounding variables into the findings.

#### MR analysis

Genetic variants as IVs were utilized to determine whether lifestyle has a causative effect on semen quality in our MR analysis. The inverse-variance weighted (IVW) method was used as the primary approach in this study^[^28^]^. Other methods as supplemental analyses were used to verify the stability and reliability of the data. The stability and reliability of the data were weighted by the methods including MR-Egger, maximum likelihood, simple median, weighted median, and penalized weighted media^[^29^]^.

Besides the primary analyses, the robustness of the results was assessed by several sensitivity analyses. The possible horizontal pleiotropic effects of the IVs were evaluated by implementing the MR-pleiotropy residual sum and outlier (MR-PRESSO) test and the MR-Egger regression. The MR-PRESSO test was used to evaluate the horizontal pleiotropic effects and calibrate outlier SNPs, with the NbDistribution set to 2000. The intercept term in MR-Egger regression was used to indicate whether directional horizontal pleiotropy was driving the results of the MR analysis^[^30^]^. Utilizing Cochran’s *Q*-test statistics, heterogeneity was quantified. Moreover, whether the causal association was influenced by a single SNP was investigated by a “leave-one-out” sensitivity analysis and the robustness of the results was assessed by funnel pot. The statistical analyses of Beta with 95%CIs for this outcome were conducted by the R version 4.4.0 by utilizing the R packages of “Two-Sample MR,” “MR-PRESSO,” and “Mendelian Randomization.”

## Result

### Basic characteristics of observational study participants

After applying the inclusion and exclusion criteria, 508 subjects entered the study. The overall characteristics of the subjects were summarized in Table 1. Of these participants, 130 (25.5%) met the diagnostic criteria for oligozoospermia, 163 (32.1%) met the diagnostic criteria for asthenozoospermia, 154 (30.3%) met the diagnostic criteria for teratozoospermia, and 210 (41.3%) met the diagnostic criteria for low acrosin activity (LAA). Except for the education level in LAA and control groups, no statistically significant difference was found between the patient and control groups in the basic characteristics.

**Table 1:** Characteristics of the study population.

Variables	Total (*n* = 375)	Oligozoospermia			Asthenozoospermia			Teratozoospermia			LAA		
		Patient (*n* = 94)	Control (*n* = 281)	*P-*value	Patient (*n* = 123)	Control (*n* = 252)	*P-*value	Patient (*n* = 109)	Control (*n* = 266)	*P-*value	Patient (*n* = 163)	Control (*n* = 212)	*P-*value
Age, (mean (SD))	32.6 (4.32)	32.9 (4.48)	32.5 (4.27)	0.488	33.0 (4.64)	32.4 (4.15)	0.219	32.4 (4.08)	32.7 (4.42)	0.451	32.1 (4.09)	33.0 (4.46)	0.052
BMI, (mean (SD))	28.8 (25.9)	27.5 (15.5)	29.4 (28.5)	0.653			0.199			0.317			0.403
Normal	165 (44.0%)	42 (44.7%)	123 (43.8%)		57 (46.3%)	108 (42.9%)		54 (49.5%)	111 (41.7%)		78 (47.9%)	87 (41.0%)	
Overweight	126 (33.6%)	34 (36.2%)	92 (32.7%)		34 (27.6%)	92 (36.5%)		35 (32.1%)	91 (34.2%)		50 (30.7%)	76 (35.8%)	
Obesity	84 (22.4%)	18 (19.1%)	66 (23.5%)		32 (26.0%)	52 (20.6%)		20 (18.3%)	64 (24.1%)		35 (21.5%)	49 (23.1%)	
Smoking (%)				0.016			< 0.001			0.001			0.006
Non-smoking	234 (62.4%)	47 (50.0%)	187 (66.5%)		61 (49.6%)	173 (68.7%)		52 (47.7%)	182 (68.4%)		89 (54.6%)	145 (68.4%)	
Former smoking	39 (10.4%)	13 (13.8%)	26 (9.25%)		12 (9.76%)	27 (10.7%)		18 (16.5%)	21 (7.89%)		16 (9.82%)	23 (10.8%)	
Current smoking	102 (27.2%)	34 (36.2%)	68 (24.2%)		50 (40.7%)	52 (20.6%)		39 (35.8%)	63 (23.7%)		58 (35.6%)	44 (20.8%)	
Alcohol consumption (%)				0.001			0.007			0.179			0.365
Non-alcohol consumption	210 (56.0%)	43 (45.7%)	167 (59.4%)		54 (43.9%)	156 (61.9%)		52 (47.7%)	158 (59.4%)		84 (51.5%)	126 (59.4%)	
Former alcohol consumption	63 (16.8%)	11 (11.7%)	52 (18.5%)		27 (22.0%)	36 (14.3%)		22 (20.2%)	41 (15.4%)		30 (18.4%)	33 (15.6%)	
Soft alcohol consumption	69 (18.4%)	25 (26.6%)	44 (15.7%)		26 (21.1%)	43 (17.1%)		22 (20.2%)	47 (17.7%)		31 (19.0%)	38 (17.9%)	
Heavy alcohol consumption	33 (8.80%)	15 (16.0%)	18 (6.41%)		16 (13.0%)	17 (6.75%)		13 (11.9%)	20 (7.52%)		18 (11.0%)	15 (7.08%)	
Coffee consumption (%)				0.001			0.139			0.02			0.907
Non-coffee consumption	273 (72.8%)	68 (72.3%)	205 (73.0%)		83 (67.5%)	190 (75.4%)		77 (70.6%)	196 (73.7%)		118 (72.4%)	155 (73.1%)	
Soft coffee consumption	75 (20.0%)	12 (12.8%)	63 (22.4%)		27 (22.0%)	48 (19.0%)		18 (16.5%)	57 (21.4%)		34 (20.9%)	41 (19.3%)	
Heavy coffee consumption	27 (7.20%)	14 (14.9%)	13 (4.63%)		13 (10.6%)	14 (5.56%)		14 (12.8%)	13 (4.89%)		11 (6.75%)	16 (7.55%)	
Education level (%)				0.273			0.174			0.719			0.013
Less than or equal to high school	96 (25.6%)	28 (29.8%)	68 (24.2%)		37 (30.1%)	59 (23.4%)		31 (28.4%)	65 (24.4%)		54 (33.1%)	42 (19.8%)	
College degree	246 (65.6%)	61 (64.9%)	185 (65.8%)		79 (64.2%)	167 (66.3%)		69 (63.3%)	177 (66.5%)		95 (58.3%)	151 (71.2%)	
More than or equal to master degree	33 (8.80%)	5 (5.32%)	28 (9.96%)		7 (5.69%)	26 (10.3%)		9 (8.26%)	24 (9.02%)		14 (8.59%)	19 (8.96%)	
Sedentary behavior (%)				<0.001			<0.001			<0.001			0.009
Sedentary time > 8 h/day	258 (68.8%)	48 (51.1%)	210 (74.7%)		66 (53.7%)	192 (76.2%)		60 (55.0%)	198 (74.4%)		100 (61.3%)	158 (74.5%)	
Sedentary time < 8 h/day	117 (31.2%)	46 (48.9%)	71 (25.3%)		57 (46.3%)	60 (23.8%)		49 (45.0%)	68 (25.6%)		63 (38.7%)	54 (25.5%)	
Sleep disturbance (%)				0.466			0.456			0.834			0.757
Seep time < 6 h/day	42 (11.2%)	13 (13.8%)	29 (10.3%)		12 (9.76%)	30 (11.9%)		13 (11.9%)	29 (10.9%)		19 (11.7%)	23 (10.8%)	
Sleep time from 6–9 h/day	321 (85.6%)	77 (81.9%)	244 (86.8%)		109 (88.6%)	212 (84.1%)		92 (84.4%)	229 (86.1%)		140 (85.9%)	181 (85.4%)	
Sleep time > 9 h/day	12 (3.20%)	4 (4.26%)	8 (2.85%)		2 (1.63%)	10 (3.97%)		4 (3.67%)	8 (3.01%)		4 (2.45%)	8 (3.77%)	

### Unhealthy lifestyle exposure and semen quality in the logistic regression model

To assess the potential link between the six unhealthy lifestyle habits (smoking, alcohol consumption, coffee consumption, sedentary behavior, obesity, and sleep disturbance) and the prevalence of low semen quality, a binary logistic regression model was employed in which risk factors were determined using the univariate analysis. Model 1 was a crude model without covariates adjusted. Model 2 was adjusted by age and education level. Of the four outcome categories, both models showed a significant difference between sedentary time <8 hours/day and sedentary time ≥8 hours/day groups with OR and 95% CI of 4.02 (2.57, 6.35), 3.68 (2.43, 5.62), 2.17 (1.33, 3.55), and 2.60 (1.81, 3.86) (*P* < 0.01) in Model 1 (all *P-*t <0.001); 3.99 (2.54, 6.33), 3.65 (2.40, 5.59), 3.61 (2.34, 5.59), and 2.70 (1.81, 4.06) (*P* < 0.001) in Model 2 (all *P-*t <0.005), respectively. Linear trend analysis revealed a significant association between the sedentary time ≥ 8 hours/day and low semen quality in the two multivariate regression models (all *P-*t < 0.05).

When oligozoospermia was set as an outcome variable, Table 2 shows that heavy alcohol consumption was significantly correlated with the elevated prevalence of oligozoospermia in the two models with OR and 95% CI of 1.72 (0.89, 3.27) (*P* < 0.05), with *P-*t < 0.05. Additionally, an increased, 3.04-fold (95% CI: 1.85–5.01, *P* < 0.001) risk of oligozoospermia for current smoking in Model 1, *P-*t < 0.001, was observed. After adjustment for covariates in Model 2, significant difference was also obtained in current smoking effects on outcome.

**Table 2:** Multivariate logistic regression analysis of the unhealthy population with low semen quality.

Variables	Oligozoospermia		Asthenozoospermia		Teratozoospermia		LAA	
	Model 1	Model 2	Model 1	Model 2	Model 1	Model 2	Model 1	Model 2
	OR (95% CI)	OR (95% CI)	OR (95% CI)	OR (95% CI)	OR (95% CI)	OR (95% CI)	OR (95% CI)	OR (95% CI)
Smoking								
Non-smoking	1 (reference)	1 (reference)	1 (reference)	1 (reference)	1 (reference)	1 (reference)	1 (reference)	1 (reference)
Former smoking	1.97 (0.85, 4.42)	1.78 (0.75, 4.15)	1.02 (0.45, 2.19)	0.95 (0.40, 2.11)	3.25 (1.56, 6.72)^b^	3.37 (1.57, 7.23)^b^	1.12 (0.55, 2.24)	0.96 (0.45, 1.98)
Current smoking	1.90 (1.07, 3.38)^a^	1.78 (0.96, 3.28)	2.37 (1.41, 3.98)^b^	2.22 (1.28, 3.88)^b^	2.31 (1.37, 3.92)^b^	2.47 (1.39, 4.40)^b^	2.10 (1.30, 3.40)^b^	1.88 (1.12, 3.18)^a^
*P*-t	0.022		0.001	0.005	<0.001	<0.001	0.003	0.021
Alcohol consumption								
Non-alcohol consumption	1 (reference)	1 (reference)	1 (reference)	1 (reference)				
Former alcohol consumption	0.65 (0.28, 1.37)	0.68 (0.29, 1.45)	1.99 (1.06, 3.71)^a^	2.07 (1.10, 3.89)^a^				
Soft alcohol consumption	1.76 (0.88, 3.48)	1.87 (0.93, 3.74)	1.73 (0.92, 3.20)	1.81 (0.96, 3.39)				
Heavy alcohol consumption	2.66 (1.16, 6.02)^a^	2.71 (1.16, 6.26)^a^	2.05 (0.90, 4.62)	1.98 (0.86, 4.51)				
*P*-t	0.011	0.008	0.021	0.022				
Coffee consumption								
Non-coffee consumption	1 (reference)	1 (reference)			1 (reference)	1 (reference)		
Soft coffee consumption	0.62 (0.28,1.26)	0.65 (0.29,1.35)			0.91 (0.47,1.68)	0.87 (0.44,1.65)		
Heavy coffee consumption	2.79 (1.15,6.79)^a^	3.01 (1.22,7.49)^a^			2.84 (1.23,6.60)^a^	2.73 (1.17,6.44)^a^		
*P*-t	0.189	0.125			0.064	0.085		
Sedentary behavior								
Sedentary time <8 h/day	1 (reference)	1 (reference)	1 (reference)	1 (reference)	1 (reference)	1 (reference)	1 (reference)	1 (reference)
Sedentary time> 8 h/day	2.57 (1.53, 4.31)^c^	2.63 (1.56, 4.44)^c^	2.75 (1.71, 4.44)^c^	2.78 (1.72, 4.51)^c^	2.17 (1.33, 3.55)^b^	2.23 (1.35, 3.66)^b^	1.80 (1.15, 2.82)^b^	1.96 (1.24, 3.13)^c^
*P*-t	<0.001	<0.001	<0.001	<0.001	0.002	0.002	0.009	0.004

*P*-t, *P* value for trend.

Model 1: unadjusted model; Model 2: adjusted for age and education level.

^a^ *P* < 0.05.

^b^ *P* < 0.01.

^c^ *P* < 0.001.

When asthenozoospermia was considered as an outcome variable, Table 2 shows that current smoking was significantly correlated with the elevated prevalence of asthenozoospermia in the two models with OR and 95% CI of 3.55 (2.26, 5.62) and 3.86 (2.39, 6.30), respectively, both with *P* < 0.01. Additionally, former alcohol consumption was significantly correlated with the elevated prevalence of asthenozoospermia in the two models with OR and 95% CI of 1.77 (1.00, 3.13) (*P* < 0.01).

When teratozoospermia was set as an outcome variable, Table 2 shows that smoking was significantly correlated with an elevated prevalence of teratozoospermia. In Model 1, the risk increased by 1.35-fold (95% CI: 1.56–6.72, *P* < 0.01) for former smoking, and by 2.31-fold (95% CI: 1.37–3.92, *P* < 0.01) for current smoking compared to non-smoking, both *P-*t < 0.001. Similarly, in Model 2, the risk increased by 4.06-fold (95% CI: 2.10–7.89, *P* < 0.001) for former smoking, and by 3.11-fold (95% CI: 1.90–5.14, *P* < 0.001) for current smoking compared to non-smoking, and the *P* for trend < 0.001. Besides, the heavy coffee consumption was significantly correlated with an elevated prevalence of teratozoospermia in both models with OR and 95% CI of 2.84 (1.23, 6.60) and 2.14 (1.28, 3.60) (*P* < 0.05), with *P* for trend reaching a significant level (*P-*t < 0.05).

When LAA was used as an outcome variable, the risk of developing LAA positively correlated with the smoking degree. The risk increased by 2.81-fold (95% CI: 1.85–4.34, *P-*t < 0.001) in Model 1, and by 2.79-fold (95% CI: 1.78–4.40, *P* < 0.05) in current smoking compared to non-smoking, with the *P* for trend significantly different (*P-*t < 0.001).

The ROC AUCs (95% CI) of Model 1 and Model 2 on oligozoospermia were 0.884 (0.846, 0.922) and 0.891 (0.854, 0.923), respectively. The ROC AUCs (95% CI) of Model 1 and Model 2 on asthenozoospermia were 0.753 (0.702, 0.804) and 0.761 (0.709, 0.811). The ROC AUCs (95% CI) of Model 1 and Model 2 on teratozoospermia were 0.779 (0.728, 0.829) and 0.789 (0.739, 0.839). The ROC AUCs (95% CI) of Model 1 and Model 2 on LAA were 0.659 (0.604, 0.713) and 0.694 (0.641, 0.747), respectively (Supplemental Digital Content Figure 1, available at: http://links.lww.com/MS9/B139). Thus, the ROC curves validated the considerable predictive performance for the two models.

### Causal relationships between seven unhealthy lifestyle habits and semen quality–related genetic aspects

For a lack of semen quality-related databases, we investigated genetic aspects of oligozoospermia, asthenozoospermia, teratozoospermia, and abnormal acrosome as outcomes. From the large-scale public summary statistical data of GWAS datasets, 43 with the genetic dates were included in the analyses (Supplemental Digital Content Table S1, available at: http://links.lww.com/MS9/B140).

**Figure 1.**
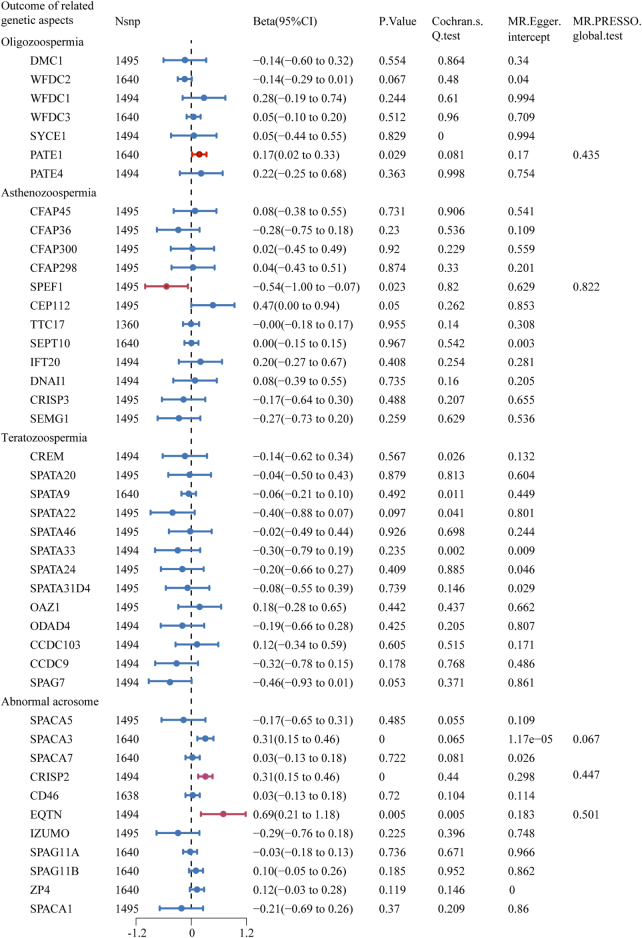
Forest plot of MR estimates between smoking and semen quality–related genetic aspects. The figure showed the IVW estimates of significantly smoking-associated semen quality–related genetic aspects (PATE1, SPEF1, CRISP2, and EQTN). The dots represent the IVW estimates, and the bars represent the 95% confidence intervals of IVW estimates.

The two-sample MR analysis revealed that there was a significant causal relationship between smoking and semen quality-related genetic aspects: prostate and testis expressed 1 (PATE1), sperm flagellar 1 (SPEF1), cysteine-rich secretory protein 2 (CRISP2), and equatorin (EQTN) (Beta = 0.17, 95% CI: 0.02–0.33, *P* = 0.029; Beta = −0.54, 95% CI: −1.00 to −0.07, *P* = 0.023; Beta = 0.31, 95% CI: 0.15–0.46, *P* <0.001; Beta = 0.69, 95% CI: 0.21–1.18, *P* = 0.005) (Fig. 1 and Supplemental Digital Content Figure S4, available at: http://links.lww.com/MS9/B139). The *P*-values of the MR-Egger intercept and MR-PRESSO global test showed no significance of directional pleiotropy (0.170 and 0.435 for PATE1; 0.629 and 0.822 for SPEF1; 0.298 and 0.447 for CRISP2; and 0.183 and 0.501 for EQTN) (Fig. 1). Except for EQTN, there was no significant heterogeneity by Cochran’s *Q* test (*P* = 0.081; 0.820; and 0.440). Although the MR analysis of sperm acrosome associated 3 (SPACA3) showed a statistical significance, the *P-*value of pleiotropy was <0.05. In addition, the results of MR Egger, simple median, maximum likelihood, penalized weighted median, and weighted median methods were similar to those of IVWs (Supplemental Digital Content Table S3, available at: http://links.lww.com/MS9/B140).

**Figure 2.**
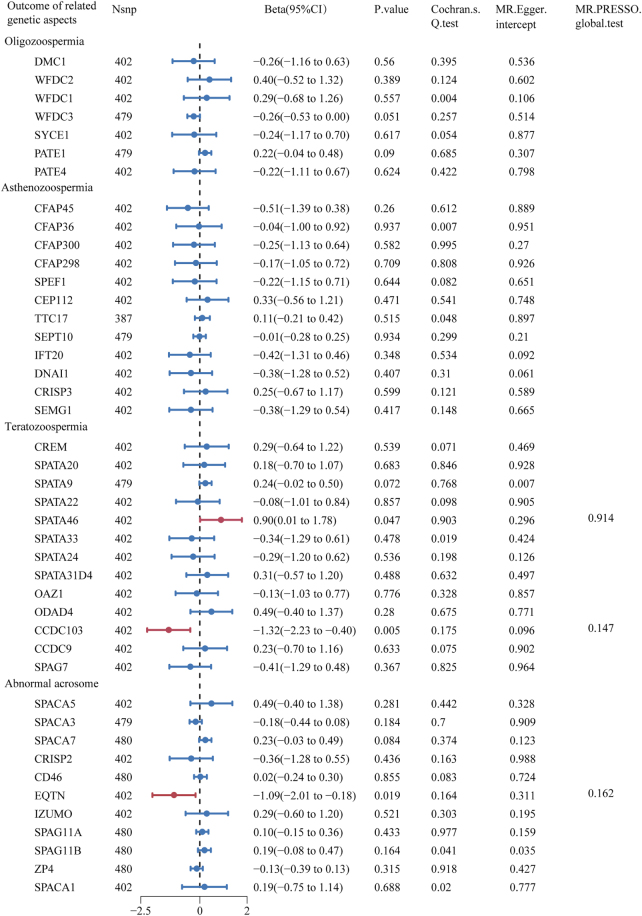
Forest plot of MR estimates between alcohol consumption and semen quality–related genetic aspects. The figure showed the IVW estimates of significantly alcohol consumption-associated semen quality–related genetic aspects (SPATA46, CCDC103, and EQTN). The dots represent the IVW estimates, and the bars represent the 95% confidence intervals of IVW estimates.

There was a significant causal relationship between alcohol consumption and semen quality-related genetic aspects: spermatogenesis associated 46 (SPATA46), and coiled-coil domain containing 103 (CCDC103), EQTN (Beta = 0.90, 95% CI: 0.01–1.78, *P* = 0.047; Beta = −1.32, 95% CI: −2.23 to −0.40, *P* = 0.005; Beta = −1.09, 95% CI: −2.01 to −0.18, *P* = 0.019) (Fig. 2 and Supplemental Digital Content Figure S4, available at: http://links.lww.com/MS9/B139). MR-Egger intercept (*P* = 0.296, 0.096, and 0.311) and MR-PRESSO global tests (*P* = 0.914, 0.147, and 0.162) showed that no significant directional pleiotropy existed for the associations of alcohol consumption and semen quality-related genetic aspects (Fig. 2). Except for EQTN, there was no significant heterogeneity by Cochran’s *Q* test (*P* = 0.903 and 0.175) (Fig. 2). Although not all sensitivity analysis showed significant association between alcohol consumption and semen quality-related genetic aspects (Supplemental Digital Content Table S4, available at: http://links.lww.com/MS9/B140), this did not affect our conclusions because sensitivity analyses present a weaker statistical performance than IVW analysis, as they were only used for measuring the consistency in the direction of the effect.

**Figure 3.**
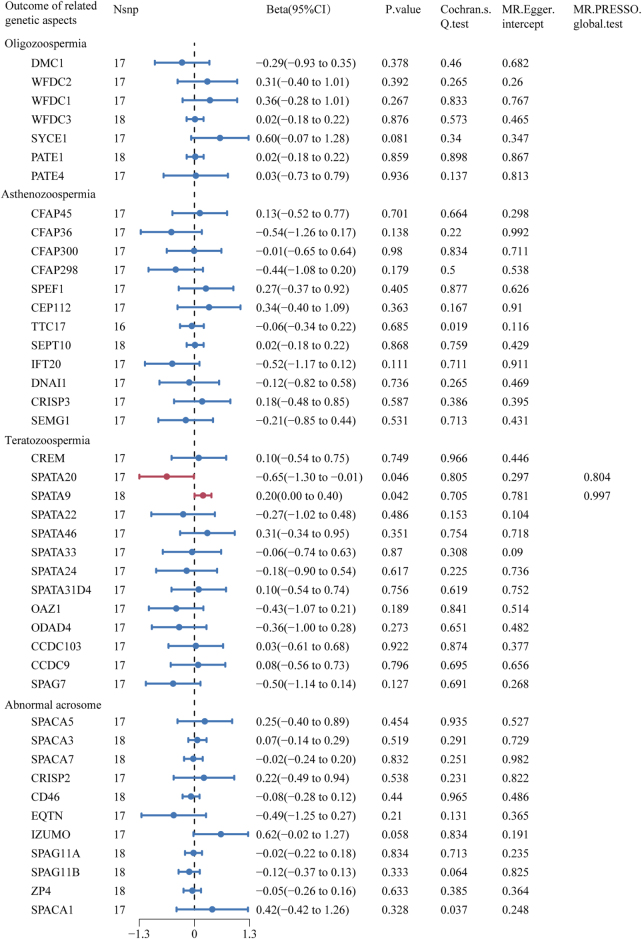
Forest plot of MR estimates between caffeine consumption and semen quality related genetic aspects. The figure showed the IVW estimates of significantly caffeine consumption-associated semen quality–related genetic aspects (SPATA20 and SPATA9). The dots represent the IVW estimates, and the bars represent the 95% confidence intervals of IVW estimates.

There was a significant causal relationship between caffeine consumption and semen quality-related genetic aspects: spermatogenesis associated 20 (SPATA20) and spermatogenesis associated 9 (SPATA9) (Beta = −0.65, 95% CI: −1.30 to −0.01, *P* = 0.046; Beta = 0.20, 95% CI: 0.00–0.40, *P* = 0.042) (Fig. 3 and Supplemental Digital Content Figure S4, available at: http://links.lww.com/MS9/B139). Moreover, neither the MR-Egger intercept (*P* = 0.297 and 0.781) nor the MR-PRESSO global tests (*P* = 0.804 and 0.997) indicated directional pleiotropy for any of these causal associations (Fig. 3). No significant heterogeneity was detected by Cochran’s *Q* test (*P* = 0.805 and 0.705) (Fig. 3). The results of the other five MR methods were similar to those of IVWs (Supplemental Digital Content Table S5, available at: http://links.lww.com/MS9/B140).

**Figure 4.**
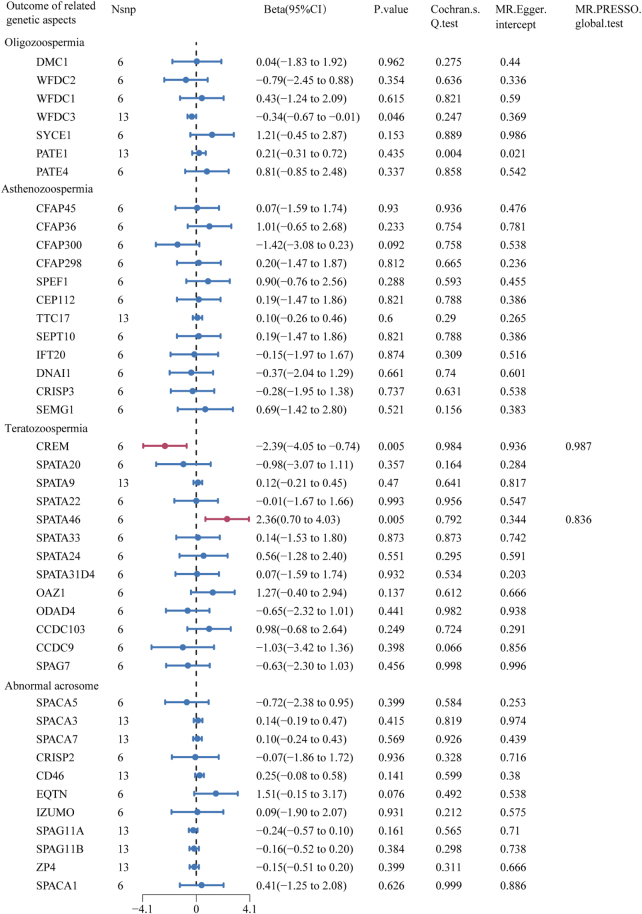
Forest plot of MR estimates between cannabis consumption and semen quality–related genetic aspects. The figure showed the IVW estimated of significantly cannabis consumption-associated semen quality–related genetic aspects (CREM and SPATA46). The dots represent the IVW estimates, and the bars represent the 95% confidence intervals of IVW estimates.

There was a significant causal relationship between cannabis consumption and semen quality-related genetic aspects: cAMP responsive element modulator (CREM), and spermatogenesis associated 46 (SPATA46) (Beta = −2.39, 95% CI: −4.05 to −0.74, *P* = 0.005; Beta = 2.36, 95% CI: 0.70–4.03, *P* = 0.005) (Fig. 4 and Supplemental Digital Content Figure S4, available at: http://links.lww.com/MS9/B139). Similar results were obtained with the use of other five MR methods (Supplemental Digital Content Table S6, available at: http://links.lww.com/MS9/B140). Moreover, both the MR-Egger intercept (*P* = 0.936 and 0.344) and MR-PRESSO global tests (*P* = 0.987 and 0.836) did not indicate directional pleiotropy for any of these causal associations (Fig. 4). No significant heterogeneity was seen by Cochran’s *Q* test (*P* = 0.984 and 0.792) (Fig. 4).

**Figure 5.**
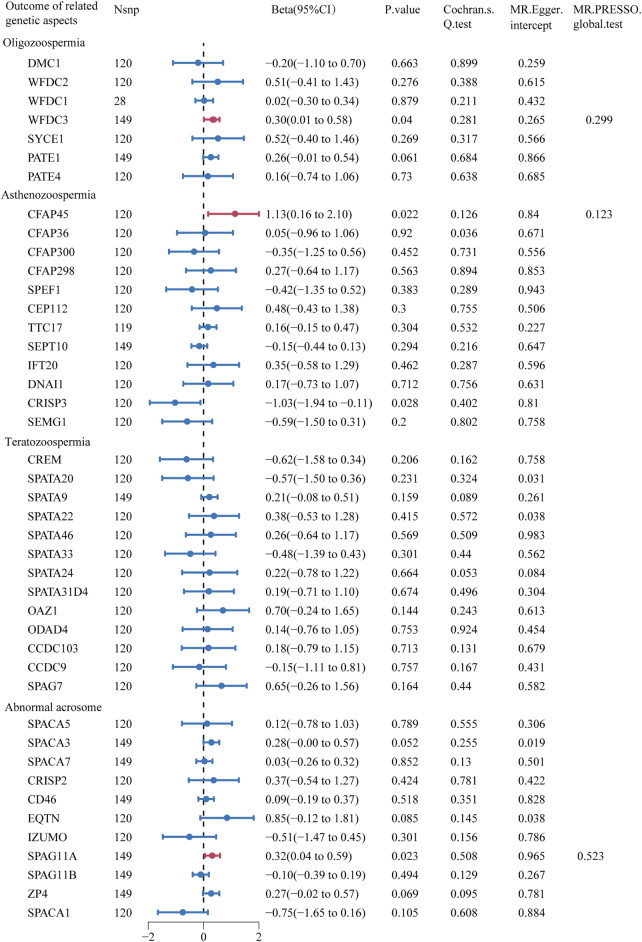
Forest plot of MR estimated between sedentary behavior and semen quality related genetic aspects. The figure showed the IVW estimates of significantly sedentary behavior-associated semen quality–related genetic aspects (WFDC3, CFAP45, and SPAG11A). The dots represent the IVW estimates, and the bars represent the 95% confidence intervals of IVW estimates.

There was a significant causal relationship between sedentary behavior and semen quality-related genetic aspects: WAP four-disulfide core domain 3 (WFDC3), cilia and flagella associated protein 45 (CFAP45), and sperm associated antigen 11A (SPAG11A)) (Beta = 0.30, 95% CI: 0.01–0.58, *P* = 0.040; Beta = 1.13, 95% CI: 0.16–2.10, *P* = 0.022; Beta = 0.32, 95% CI: 0.04–0.59, *P* = 0.023) (Fig. 5 and Supplemental Digital Content Figure S4, available at: http://links.lww.com/MS9/B139). Moreover, neither the MR-Egger intercept (*P* = 0.265, 0.840, and 0.965) nor the MR-PRESSO global tests (*P* = 0.299, 0.123, and 0.523) indicated directional pleiotropy for any of these causal associations (Fig. 5). No significant heterogeneity was detected by Cochran’s *Q* test (*P* = 0.281, 0.126, and 0.508) (Fig. 5). Similar results were acquired with the use of the other five MR methods (Supplemental Digital Content Table S7, available at: http://links.lww.com/MS9/B140).

According to the two-sample MR analysis, we estimated that there was no causal link between obesity (Supplemental Digital Content Figure S2, available at: http://links.lww.com/MS9/B139), sleep disturbance (Supplemental Digital Content Figure S3, available at: http://links.lww.com/MS9/B139), and semen quality-related genetic aspects. All of the sensitivity analysis results were consistent with those of IVW methods (Supplemental Digital Content Table S8, available at: http://links.lww.com/MS9/B140 and Supplemental Digital Content Table S9, available at: http://links.lww.com/MS9/B140).

We also performed a “leave-one-out” sensitivity analysis to identify potentially influential SNPs (Supplemental Digital Content Figure S5, available at: http://links.lww.com/MS9/B139 and Supplemental Digital Content Figure S6, available at: http://links.lww.com/MS9/B139). The funnel plots were symmetrical (Supplemental Digital Content Figure S7, available at: http://links.lww.com/MS9/B139), indicating the absence of observable horizontal pleiotropy for any of the outcomes.

Similarly, the scatter plots of 14 semen quality-related proteins’ levels showed similar causal estimates for IVW, MR Egger, and weighted median methods (Supplemental Digital Content Figure S4, available at: http://links.lww.com/MS9/B139), indicating that the MR results were accurate.

## Discussion

In this study, we combined a cross-sectional investigation using the clinical data of our center and SNP-based genetic analyses to explore the causal association between seven unhealthy lifestyle and semen quality-related genetic aspects using the MR framework. The results of observational study indicated that smoking, heavy alcohol and coffee consumption, and sedentary time ≥8 hours/day significantly related to the risk of low semen quality. In MR study, we found robust evidence supporting a causal effect of smoking, alcohol, caffeine and cannabis consumption, and sedentary behavior on genetic aspects related to oligozoospermia (WFDC3 and PATE1), asthenozoospermia (CFAP45 and SPEF1), teratozoospermia (CREM, SPATA20, SPATA9, SPATA46, and CCDC103), and abnormal acrosome (CRISP2, EQTN, and SPAG11A). IVW, along with five conventional MR methods, consistently lacked support for causation between obesity or sleep disturbance and semen quality–related genetic aspects.

Most of the previous longitudinal studies on smoking suggested its detrimental effect on semen quality^[^6,31^]^. The results of the current study consistently support this view. While results of experimental studies were not conclusive regarding the direct effect of smoking on the semen quality, some studies indicated the presence of indirect pathways leading to the low semen quality. For example, smoking may promote oxidative stress, alter sperm DNA methylation patterns, and lead to cell death^[^32^]^. Although one MR study suggested that having ever smoked was unrelated to infertility in men^[^33^]^, the detrimental effect of smoking on semen quality could not be excluded. Since infertility is a couple-dependent function involving both the male and female sides, the effect of smoking on semen quality may not be fully manifested. Our MR study revealed that smoking may cause oligozoospermia (PATE1), asthenozoospermia (SPEF1), and abnormal acrosome (CRISP2, EQTN) by affecting related genetic aspects. For example, EQTN was localized in the acrosomal membrane; and when EQTN^−/−^ male mice mated with wild type female mice, a significant reduction in the number of sperms attached to the zona-free oocyte as well as a reduction in the fertility rate were observed^[^34^]^. Thus, smoking may reduce semen quality by causing those related genetic aspects.

Alcohol is mainly oxidized by the liver, producing acetaldehyde and acetate, which are carcinogenic and toxic metabolites. Numerous clinical studies have conducted that alcohol abuse affected semen quality and male reproductive capacity (the reproductive hormonal regulation, semen quality, and sexual dysfunction)^[^7,8,35^]^. Our cross-sectional investigation found that patients with heavy alcohol consumption had a higher morbidity of oligozoospermia than those without alcohol consumption. Data from an animal study showed that an alcohol-rich diet affected testicular function, leading to negative consequences on the semen quality^[^36^]^. Our MR study indicated a causal link between alcohol consumption and teratozoospermia (SPATA46, CCDC103)/abnormal acrosome (EQTN)-related genetic aspects.

Caffeine is found in coffee, soft drinks, tea, and chocolate. Male coffee/caffeine consumption was reported to be associated with high levels of testosterone and sex hormone–binding globulin^[^9,37^]^. It was hypothesized that caffeine alters the glycolytic and oxidative profile of Sertoli cells, interfering with male’s reproductive potential^[^38^]^. Moreover, Schmid *et al* reported that men with substantial daily caffeine consumption increased sperm DNA damage associated with double-strand DNA breaks^[^39^]^. Our cross-sectional and MR studies revealed that caffeine consumption may cause a low semen quality.

Priskorn *et al* reported that time of sedentary behavior (watching television) was associated with lower sperm counts, increased follicle-stimulating hormone, decreased testosterone hormone, and decreased testosterone/luteinizing hormone ratio^[^13^]^. Moreover, a study by Kamil *et al* revealed that sedentary job increased the risk of sperm DNA damage^[^40^]^. We found that the group with the sedentary time ≥8 hours/day had a lower semen quality than control group. The pathological mechanism could be related to testicular heat stress that results in the failure of sperm chromatin remodeling during spermiogenesis. Our MR study indicated a causal link between sedentary behavior and related genetic aspects of oligozoospermia (WFDC3), asthenozoospermia (CRISP3), and abnormal acrosome (SPAG11A).

Previous research supported a role for cannabis in the reduction of sperm count and concentration, the induction of sperm cell morphological abnormalities, the reduction of sperm motility and viability, and the inhibition of capacitation and fertilizing capacity^[^11,41^]^. Animal models demonstrated the detrimental effects of cannabis for testicular atrophy, reducing libido and sexual function. However, to our knowledge these results have not yet been replicated in human studies^[^42,43^]^. For policy reasons, cannabis is banned in China, and our cross-sectional study could not analyze the relationship between cannabis consumption and semen quality. Our results of MR analysis support a causal relationship between cannabis and teratozoospermia (CREM and SPATA46). At present, the mechanism by which cannabis affects semen quality is unclear, and the MR results may provide a direction for future studies.

The negative effect of obesity on male fertility is well known, likely through alteration in the hypothalamic–pituitary–gonadal axis, the disruption of testicular steroidogenesis, and metabolic dysregulation involving insulin, cytokines, and adipokines^[^44,45^]^. Importantly, obesity and its underlying mediators cast negative impacts on semen parameters, including sperm concentration, motility, and normal morphology. Moreover, obesity inhibited chromatin condensation to increase DNA fragmentation, cell apoptosis, and epigenetic changes that can be transferred to offspring^[^14^]^. However, our cross-sectional and MR analyses did not find evidence for the putative causal effect of obesity on semen quality-related genetic aspects. The potential explanation for this discrepancy might be that observational studies are subject to the inherent defects of residual confounding and reverse effects.

The frequency of sleep disturbance has increased in the industrialized world during the past few decades. A cross-sectional study reported that men with a high level of sleep disturbance (score > 50) had a 29% lower adjusted sperm concentration and 1.6% fewer morphologically normal spermatozoa than men with a sleep score of 11–20^[^46^]^. The change might be attributed to hormonal and metabolic dysregulation. No association between sleep disturbance and semen quality-related genetic aspects were found in our cross-sectional and MR analyses. This might be due to the fact that the heterogeneity and polygenicity of sleep disturbance has not yet been fully explored by the presently available GWAS, which covers only 16 SNPs.

The limitations of our study should be noted. First, our results were based on genetic data from European populations, which limited their extrapolation to other populations. Second, due to the lack of semen quality-related databases, we investigated genetic aspects of semen quality as outcomes. Many key semen quality genetic aspects such as dynein axonemal heavy chain family^[^47,48^]^ were not included in the study for the lack of GWAS databases. This will limit the statistical power and introduce variations into the MR estimates. Besides, the single center and quite short time cross-sectional study may cause data bias.

In summary, the present MR study supported smoking, alcohol, caffeine and cannabis consumption, and sedentary behavior as causal risk factors for semen quality-related genetic aspects. The previously observed links might be confounded by underlying factors and require further validation. Our study found that there might be no causal relationship between obesity or sleep disturbance and semen quality-related genetic aspects. Meanwhile, the research method is also suitable to explore the causal relationships between unhealthy lifestyle and other diseases.

## Data Availability

The data presented in this study are available on reasonable request from the corresponding author.
